# Vitamin D levels are prognostic factors for connective tissue disease associated interstitial lung disease (CTD-ILD)

**DOI:** 10.18632/aging.102890

**Published:** 2020-03-12

**Authors:** Yujuan Gao, Qi Zhao, Xiaohua Qiu, Yi Zhuang, Min Yu, Jinghong Dai, Hourong Cai, Xin Yan

**Affiliations:** 1 Department of Respiratory and Critical Care Medicine, Nanjing Drum Tower Hospital, The Affiliated Hospital of Nanjing University Medical School, Nanjing 210008, Jiangsu, China

**Keywords:** Vitamin D, connective tissue disease associated interstitial lung disease, prognosis

## Abstract

Objective: Vitamin D deficiency was associated with CTD-ILD and reduced lung function. We sought to confirm that lower Vitamin D level would be related to shorter survival times.

Results: The CTD-ILD patients had lower Vitamin D level(P<0.05). Among patients with CTD-ILD who have improved lung function after treatment, elevation of Vitamin D level was positively associated with ΔFVC (%), ΔFEV1(%) and ΔDLCO-SB (%). The median survival time of patients with high serum 25(OH)D level was significantly longer than the patients with low 25(OH)D level group (16.5 months vs14.0 months, P=0.007). The Vitamin D was identified as an independent prognostic factor with a hazard ratio of 0.869 (95% CI 0.772-0.977, P =0.019).

Conclusions: Vitamin D level was lower in patients with CTD-ILD and associated with poor prognosis. Continuous levels of Vitamin D may be an important serum biomarker of prognosis.

Methods: 85 CTD-ILD patients, 71 Idiopathic pulmonary fibrosis (IPF) patients and 78 healthy control patients were included in the study. In the subgroup analysis, the CTD-ILD patients were divided into anti-MDA5 antibody-positive group and anti-MDA5 antibody-negative group according to the serum autoantibodies results. The survival analysis evaluated effect of Vitamin D level on disease prognosis.

## INTRODUCTION

Interstitial lung disease (ILD) is strongly associated with connective tissue disease (CTD) including polymyositis/dermatomyositis (PM/DM), systemic lupus erythematosus (SLE), rheumatoid arthritis (RA), Sjögren’s syndrome, and so on [1]. Although it reported that the median survival time for CTD-ILD patients was roughly 6.5 years, and the mortality rate due to CTD attributable to ILD was approximately 123.6 per 1000 person years [2, 3], but the progression and prognosis of CTD-ILD varies widely between individuals [4]. In general, the poor prognosis of ILD was reported to correlate with the dyspnea score, pulmonary function degree [5, 6], patterns of high-resolution computed tomography (HRCT) scans [7, 8]. Limited by the patient's cooperation, we try to find some reliable and less expensive serum biomarkers which are also easy to detect. Although it has been showed that increased serum surfactant protein D (SP-D) and Krebs von den Lungen-6 (KL-6) concentrations were associated with the decline of forced vital capacity (FVC) and the severity of interstitial pneumonia for patients with CTD-ILD [9–11], they have not been widely used in clinical practice. There was no disease-specific biomarker in CTD-ILD that can predict disease progression and prognosis.

Vitamin D is recognized as a steroid hormone with immunomodulatory properties which also regulates bone development, calcium homeostasis [12]. 25-hydroxyVitamin D_3_ (25(OH)D) is the last metabolic product of Vitamin D. Recent studies suggested that Vitamin D also plays a role in lung tissue remodeling, keeping lung function and immune system modulation [13]. The serum 25(OH)D was lower in patients with RA, SLE and undifferentiated connective tissue disease (UCTD), compared with healthy control patients [14–17]. But it has been suggested that corticosteroid usage reduces 25(OH)D levels through increased consumption [18]. A few studies showed that Vitamin D deficiency was associated with CTD-ILD and reduced lung function [13]. However, there was no study investigating whether Vitamin D deficiency correlated with shorter survival times. In this study, we evaluate the serum Vitamin D levels in patients with CTD-ILD, IPF and the healthy person to explore the hypothesis which the Vitamin D levels in patients with CTD-ILD are lower and can predict the prognosis.

## RESULTS

### Subject characteristics

**Figure 1:**
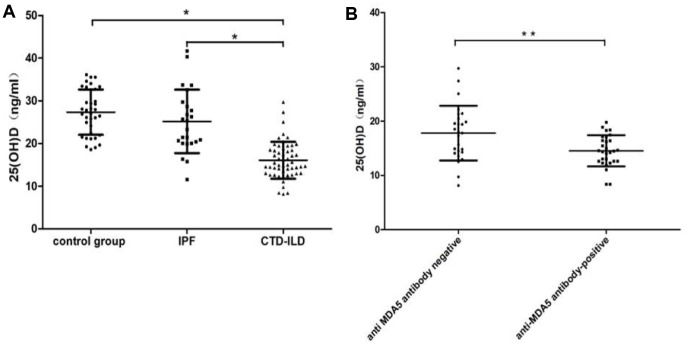
**The level of 25(OH)D in the healthy control, IPF and CTD-ILD.** (**A**) The mean level of 25(OH)D was 16.06±4.33 ng/ml in the CTD-ILD group, 25.18±7.43 ng/ml in the IPF group and 27.33±5.30 ng/ml in the control group. The serum 25(OH)D levels were obviously lower in patients with CTD-ILD compared with the IPF group (P < 0.05) and the control group (P < 0.05). (**B**) The CTD-ILD groups were divided into two subgroups: anti-MDA5 antibody-positive and anti-MDA5 antibody-negative groups. A statistically significant difference in vitamin D levels was found in the two subgroups(P=0.006).

Clinical characteristics and laboratory data of the cohorts are shown in Table 1. Two hundred and thirty-two patients were included in the study (76 healthy adults, 71 patients with IPF and 85 patients with CTD-ILD). Corticosteroids have not been used in all patients with CTD-ILD. The mean age of the control was 53.41±6.52 years, the mean age of the IPF and CTD-ILD groups were 61.24±5.76 years and 52.73±8.71 years respectively. There were no sex differences between the groups. The CTD-ILD subjects were more likely to have higher erythrocyte sedimentation rate (ESR), lactate dehydrogenase (LDH) and creatine kinase (CK) levels (all *P*<0.05). The ESR of CTD- ILD group was 33.00±7.76 mm/h whereas the control group was 13.25±4.05 mm/h, the IPF group was 15.34±5.74 mm/h. Similarly, the serum CK and LDH levels were significantly elevated in CTD-ILD patients (CK was 294.53±26.54IU/ml and LDH was 662.24±37.65IU/ml). In addition, the mean CD4+ T cells counts in the CTD-ILD group was markedly lower than that in the IPF group and the healthy control group(0.44±0.25×10^9^/L, 0.79±0.29×10^9^/L and 0.88±0.33×10^9^/L, respectively, *P*<0.05). Whereas the serum levels of CRP did not differ between the three groups at the first medical examination. As shown in Figure 1A, the mean level of 25(OH)D was 16.06±4.33 ng/ml in the CTD-ILD group, 25.18±7.43 ng/ml in the IPF group and 27.33±5.30 ng/ml in the control group. The serum 25(OH)D levels were obviously lower in patients with CTD-ILD compared with the IPF group (*P*<0.05) and the control group (*P*<0.05). Notably, 25(OH)D levels were not significantly lower for IPF patients compared to the healthy controls.

**Table 1:** Demographics and laboratory data of all subjects.

**Variable**	**Control (n=76)**	**IPF(n=71)**	**CTD-ILD(n=85)**
**Age, y, mean±SD**	53.41±6.52	61.24±5.76a	52.73±8.71b
**Men, No. (%)**	42(55.26)	39(54.92)	49(57.64)
**Smoker, No. (%)**	22(28.94)	20(28.16)	23(27.05)
**T2DM history, No. (%)**	7(9.21)	7(9.85)	8(9.41)
**Hypertension history, No. (%)**	8(10.52)	8(11.26)	10(11.76)
**CRP(mg/L)**	14.97±6.15	15.34±5.74	15.13±6.42
**ESR(mm/h)**	13.25±4.05	14.17±4.23	33.00±7.76 a, b
**LDH(IU/L)**	151.54±13.27	153.73±15.19	662.24±37.65 a, b
**CK(IU/L)**	83.62±11.62	85.07±10.07	294.53±26.54 a, b
**CD4+ T cells (×10^9^/L)**	0.88±0.33	0.79±0.29	0.44±0.25 a, b
**25(OH)D(ng/ml)**	27.33±5.30	25.18±7.43	16.06±4.33a, b

### Vitamin D Levels and CTD-ILD

The CTD-ILD groups were divided into two subgroups: anti-MDA5 antibody-positive and anti-MDA5 antibody-negative groups. Anti-MDA5 antibody has been reported to be a poor prognostic marker for dermatomyositis (DM) and clinically amyopathic dermatomyositis (CADM). The 5-year survival rate of patients with anti-MDA5 antibodies was only 56% [19]. A statistically significant difference in Vitamin D levels was found in the two subgroups, anti-MDA5 positive group vs anti-MDA5 negative group (14.52±2.86 ng/ml, n=29 vs. 17.79±5.04 ng/ml, n=56, *P*=0.006) (Figure 1B).

### The elevation of Vitamin D concentrations correlates with the improvement of pulmonary function

In the CTD-ILD group, 30 patients were included in the further analyses for before and after treatment, who received a combination of glucocorticoid and immunosuppressive therapy. A higher Vitamin D level in the patients after treatment existed than in the patients before treatment (22.42±5.27 ng/ml vs 17.21±4.99 ng/ml, *P*<0.05). Table 2 shows the variation in the pulmonary function of the samples during the therapy. Baseline lung function was altered when serum Vitamin D was increased, increasing from 58.23%±13.29% to 70.43%±16.32 % for FVC predicted, from 61.13%±15.50% to 73.66%±19.85% for FEV1 predicted and from 55.00%±16.18% to 62.42%±18.11% for DLCO-SB predicted.

**Table 2:** Comparison of vitamin D and lung function in patients with CTD-ILD before and after treatment.

**Variable**	**before treatment (n=30)**	**after treatment (n=30)**
FVC predicted (%)	58.23±13.29	70.43±16.32a
FEV1 predicted (%)	61.13±15.50	73.66±19.85a
DLCO-SB predicted (%)	55.00±16.18	62.42±18.11a
25(OH)D(ng/ml)	17.21±4.99	22.42±5.27a

**Figure 2:**
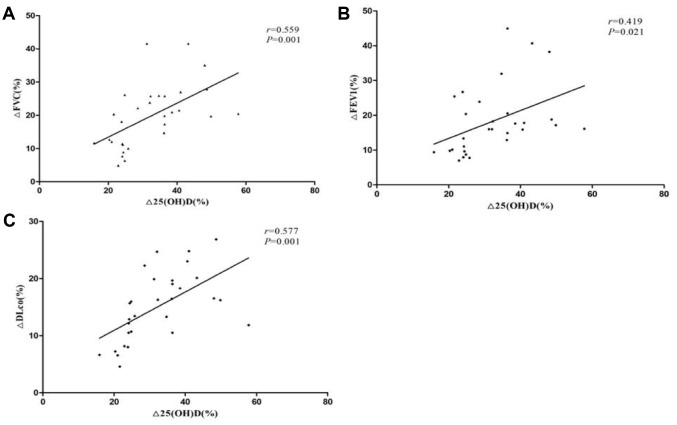
**Correlation between vitamin D and lung function changes in patients with CTD-ILD.** (**A**) The Δ25(OH)D(%) was positively correlated with ΔFVC(%) (r=0.559, P=0.001); (**B**) The Δ25(OH)D(%) was positively correlated with ΔFEV1(%) (r=0.559, P=0.001); (**C**) The Δ25(OH)D(%) was positively correlated with ΔDLCO-SB(%)(r = 0.559, P=0.001).

We further defined the percentage change of after treatment and before treatment in 25(OH)D level, FVC, FEV1 and DLCO-SB as Δ25(OH)D(%), ΔFVC(%), ΔFEV1(%) and ΔDLCO-SB(%). Spearman's rank correlation coefficients were evaluated for correlation between changes of serum Vitamin D level and the pulmonary function tests. As shown in Figure 2, the Δ25(OH)D(%) was positively correlated with ΔFVC(%), ΔFEV1(%) and ΔDLCO-SB(%). (r = 0.559, *P* = 0.001, r = 0.559, *P* = 0.001, r = 0.559, *P* = 0.001, respectively), which indicated that increased Vitamin D levels after treatment may suggest an improvement in lung function and a better condition.

### Vitamin D Levels and the survival times of patients with CTD-ILD

**Figure 3:**
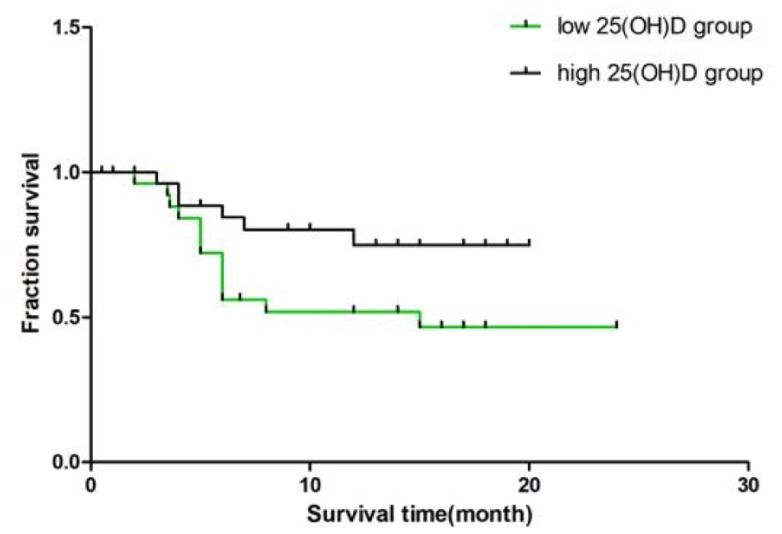
**Survival of CTD-ILD patients in high 25(OH)D level group and low 25(OH)D level group.** Using a median of serum 25(OH)D (14.98) as a standard, 85 patients with CTD-ILD were divided into high-level and low-level groups. The median survival time of patients with high serum 25(OH)D level in was 16.5 months (95%CI 14.6~18.4 months), significantly longer than the patients with low-level 25(OH)D level group (14.0 months, 95%CI 11.1 to 16.9 months) (P=0.007).

Kaplan-Meier survival curves of the patients with CTD-ILD stratified by a median of serum 25(OH)D level with a cutoff line of 14.98 ng/ml. As shown in Figure 3, 85 patients with CTD-ILD were divided into high-level and low-level groups. The median survival time of patients with high serum 25(OH)D level was 16.5 months (95%CI 14.6~18.4 months), significantly longer than the patients with low-level 25(OH)D level group (14.0 months, 95%CI 11.1 to 16.9 months) (*P*=0.007).

The results of the multivariate Cox proportional hazard model are listed in Table 3. In the multivariate Cox regression model, the Vitamin D was identified as an independent prognostic factor with a hazard ratio of 0.869 (95CI 0.772-0.977, *P* =0.019). Additionally, when the age, gender, history of smoking, hypertension and type 2 diabetes, the serum concentration of CK, LDH, CRP and ESR were adjusted, the adjusted hazard ratio for serum 25(OH)D was 0.730 (95% confidence interval 0.578-0.992, *P* = 0.008) (Table 3).

**Table 3:** Effect of serum 25(OH)D level on total survival time in Cox regression model.

**Model**	**B**	**SE**	**Wald x^2^**	**HR**	**HR(95%CI)**	**P**
1	-0.141	0.060	5.493	0.869	0.772-0.977	0.019
2	-0.314	0.119	6.972	0.730	0.578-0.992	0.008

Model 1: Unadjusted

Model 2: age, gender, history of smoking, hypertension and type 2 diabetes, the serum concentration of CK, LDH, CRP and ESR were adjusted.

## DISCUSSION

The challenge of treatment and poor prognosis of CTD-ILD are higher than CTD without ILD. It has been reported that Vitamin D deficiency was observed among those subjects with CTD especially in the CTD-ILD patients, and reduced serum 25(OH)D level was significantly associated with reduced lung function in the patient with CTD-ILD [12–15, 17]. The subjects in previous study were more likely to have received corticosteroid, which was doubtful whether Vitamin D deficiency was related to corticosteroid usage. In our study, 85 patients diagnosed CTD-ILD who did not use corticosteroid before admission were enrolled, 71 patients diagnosed IPF and 78 healthy persons as the control groups were reviewed simultaneously. As expected, the serum 25(OH)D levels were obviously lower in patients with CTD-ILD compared with the IPF group and the healthy group (Table 1, *P*<0.05). There was no significant difference in Vitamin D levels between the patients with IPF and the healthy controls, which suggested that Vitamin D deficiency is more associated with autoimmune diseases associated with CTD. Interestingly, the mean CD4+ T cells counts in the CTD-ILD group was markedly lower than the other two groups(*P*<0.05). It has been showed that 25(OH)D can inhibited T-cell proliferation [20] and low Vitamin D levels up-regulated the expression of autophagy related genes and reduced the counts of T-cell subsets in active SLE [21]. Some studies have confirmed that T lymphocytes could express Vitamin D receptor, Vitamin D could reduce secretion of cytokines via Vitamin D receptor [20]. The relationship between Vitamin D and the immune system in connective tissue disease, especially in CTD-ILD deserves further investigation. In addition, the serum ESR, CK and LDH levels were significantly elevated in CTD-ILD patients(all *P*<0.05), which probably due to the selective bias caused by more than one-third of CTD patients(29/85) diagnosed myositis with positive anti-MDA5 were included. Anti-MDA5 antibody has been reported to be a poor prognostic marker for DM and CADM. The 5-year survival rate of patients with anti-MDA5 antibodies was only 56% [19]. Subgroup analysis found that anti-MDA5 positive group had lower Vitamin D levels than the anti-MDA5 negative group (*P*=0.006), which indicated the worse the prognosis, the lower the vitamin levels.

We have known that Vitamin D deficiency is associated with reduced lung function in CTD-ILD patients, however, few studies have focused on changes of Vitamin D levels before and after treatment when lung function altered. Our results indicated that the elevation of Vitamin D concentrations positively correlates with the improvement of pulmonary function including ΔFVC(%), ΔFEV1(%) and ΔDLCO-SB(%). The exact mechanism regarding the effect of Vitamin D on lung function is unknown. There were several proposed mechanisms may explain the contribution of Vitamin D deficiency to impairing the lung function. Vitamin D works mainly through Vitamin D receptor (VDR) expressed on various inflammatory and structural cells. Vitamin D has been shown to inhibit TGF-b signaling and reduce TGF-b-mediated fibroblast proliferation [22]. It has also been shown to prevent the expression of several matrix metalloproteinases in monocytes and alveolar macrophages, takes part in the airway remodeling in chronic obstructive pulmonary disease [23, 24].

Although it has been reported that Vitamin D supplementation might be an effective therapeutic candidate for the control of autoimmune processes especially in rheumatic diseases, but our results only suggested that Vitamin D levels increased when the condition improved in the patients who did not have additional Vitamin D supplements. Moreover, Kaplan-Meier survival curves of the patients with CTD-ILD indicated that high-level Vitamin D group had longer survival time than low-level group. The results of multivariate Cox analysis showed that lower Vitamin D levels predict worse prognosis. Therefore, the baseline serum 25(OH)D levels are related to prognosis and Δ25(OH)D may be sensitive markers in predicting CTD-ILD progression, however, this hypothesis needs to increase the sample size for further confirmation.

This study still has several limitations. First, we know that international definition of Vitamin D insufficiency and deficiency were serum 25(OH)D values less than 30 ng/mL and 20 ng/mL, respectively. But Vitamin D deficiency in the Chinese population is a widespread health problem. Our study showed that the mean level of 25(OH)D was 27.33±5.30 ng/ml in the healthy control group and only 16.06±4.33 ng/ml in the CTD-ILD group. There was no control group with normal Vitamin D level for survival analysis. Second, to determine the correlation between changes of serum Vitamin D level and the pulmonary function tests, we analyzed the serum Vitamin D level in 30 CTD-ILD patients before and after treatment. The results showed that the Δ25(OH)D(%) was positively correlated with ΔFVC(%), ΔFEV1(%) and ΔDLCO-SB(%), which made us assume that Δ25(OH)D may be sensitive markers in predicting CTD-ILD progression, however, this hypothesis requires analysis of a larger sample size to support.

In conclusion, this is the first study on the relationship between Vitamin D levels and survival analysis in patients with CTD-ILD, suggesting that the changes in Vitamin D levels during treatment may predict disease progression. Continuous levels of Vitamin D may be an important serum biomarker of prognosis.

## MATERIALS AND METHODS

This study is a retrospective clinical study, which was approved by the Ethics Committee of Nanjing Drum Tower Hospital of Medical School of Nanjing University (No.31/93, 84/93, 29/01) and waived the consent. A total of 156 patients with ILD between December 2016 and December 2017 in Department of Respiratory Medicine, Nanjing Drum Tower Hospital Affiliated to Medical School of Nanjing University were included in the study. ILD was diagnosed on the basis of clinical presentation, physical examination, pulmonary function tests and HRCT images. 85 patients diagnosed CTD-ILD who met the American College of Rheumatology criteria [25], 71 patients diagnosed IPF by An Official ATS/ERS/JRS/ALAT Clinical Practice Guideline [26] and 78 healthy persons as the control group. We further used the classification of autoantibodies to define patients as anti-MDA5 antibody-positive and anti-MDA5 antibody-negative groups. The clinical data included gender, age, smoking history, chronic disease history, pulmonary function tests and laboratory findings were obtained from medical records when the serum samples were obtained. It is worth mentioning that all patients with CTD-ILD did not use corticosteroids or other immunosuppressant when we first collected Vitamin D data.

### Detection of vitamin D

Serum 25(OH)D concentrations were assayed by Roche CSE170 automatic electrochemiluminescence immunoassay analyzer at Clinical Testing Center in Nanjing Drum Tower Hospital Affiliated to Medical School of Nanjing University. The reagents were purchased from Roche (Switzerland, Lot Number: 174625). The normal values of serum 25(OH)D are from 30ng/mL to 35 ng/mL.

### Statistical analysis

Continuous data were expressed as median (range) and binary data were expressed as number (percentage). Either the chi-square test or Fisher's exact test was used as appropriate for comparing proportions. All P values corresponded to two- sided tests and statistical significance was defined as a P value of 0.05. The survival time was calculated from the date of first admission to our hospital for ILD to the last visit or death. Overall survival was evaluated by the Kaplan-Meier method using the log-rank test. Variables found to be associated at a P value of 0.05 in the unadjusted analysis and those considered important a priori were considered for inclusion in multivariate regression models. The final multivariate models were chosen using stepwise backward deletion. All analyses were performed with SPSS statistical software version 9.2 (Stata Corp; College Station, Texas).

## References

[1] Vij R, Strek ME. Diagnosis and treatment of connective tissue disease-associated interstitial lung disease. Chest. 2013; 143:814–24. 10.1378/chest.12-0741 23460159 PMC3590889

[2] Demoruelle MK, Mittoo S, Solomon JJ. Connective tissue disease-related interstitial lung disease. Best Pract Res Clin Rheumatol. 2016; 30:39–52. 10.1016/j.berh.2016.04.006 27421215

[3] Suzuki A, Kondoh Y, Fischer A. Recent advances in connective tissue disease related interstitial lung disease. Expert Rev Respir Med. 2017; 11:591–603. 10.1080/17476348.2017.1335600 28544856

[4] Navaratnam V, Ali N, Smith CJ, McKeever T, Fogarty A, Hubbard RB. Does the presence of connective tissue disease modify survival in patients with pulmonary fibrosis? Respir Med. 2011; 105:1925–30. 10.1016/j.rmed.2011.08.015 21924888

[5] Khanna D, Mittoo S, Aggarwal R, Proudman SM, Dalbeth N, Matteson EL, Brown K, Flaherty K, Wells AU, Seibold JR, Strand V. Connective tissue disease-associated interstitial lung diseases (ctd-ild) - report from omeract ctd-ild working group. J Rheumatol. 2015; 42:2168–71. 10.3899/jrheum.141182 25729034 PMC4809413

[6] Solomon JJ, Fischer A. Connective tissue disease-associated interstitial lung disease: A focused review. J Intensive Care Med. 2015; 30:392–400. 10.1177/0885066613516579 24371251

[7] Enomoto Y, Nakamura Y, Colby TV, Johkoh T, Sumikawa H, Nishimoto K, Yoshimura K, Matsushima S, Oyama Y, Hozumi H, Kono M, Fujisawa T, Enomoto N, et al. Radiologic pleuroparenchymal fibroelastosis-like lesion in connective tissue disease-related interstitial lung disease. PLoS One. 2017; 12:e0180283. 10.1371/journal.pone.0180283 28666014 PMC5493376

[8] Tanizawa K, Handa T, Kubo T, Chen-Yoshikawa TF, Aoyama A, Motoyama H, Hijiya K, Yoshizawa A, Oshima Y, Ikezoe K, Tokuda S, Nakatsuka Y, Murase Y, et al. Clinical significance of radiological pleuroparenchymal fibroelastosis pattern in interstitial lung disease patients registered for lung transplantation: a retrospective cohort study. Respir Res. 2018; 19:162. 10.1186/s12931-018-0860-6 30165854 PMC6117972

[9] Al-Salmi QA, Walter JN, Colasurdo GN, Sockrider MM, Smith EO, Takahashi H, Fan LL. Serum KL-6 and surfactant proteins A and D in pediatric interstitial lung disease. Chest. 2005; 127:403–07. 10.1378/chest.127.1.403 15654008

[10] Suematsu E, Miyamura T, Shimada H, Nakao R, Yamamoto M. [Assessment of serum markers KL-6 and SP-D for interstitial pneumonia associated with connective tissue diseases]. Ryumachi. 2003; 43:11–18. 12692985

[11] Yamakawa H, Hagiwara E, Kitamura H, Yamanaka Y, Ikeda S, Sekine A, Baba T, Okudela K, Iwasawa T, Takemura T, Kuwano K, Ogura T. Serum KL-6 and surfactant protein-D as monitoring and predictive markers of interstitial lung disease in patients with systemic sclerosis and mixed connective tissue disease. J Thorac Dis. 2017; 9:362–71. 10.21037/jtd.2017.02.48 28275485 PMC5334095

[12] van Etten E, Decallonne B, Mathieu C. 1,25-dihydroxycholecalciferol: endocrinology meets the immune system. Proc Nutr Soc. 2002; 61:375–80. 10.1079/PNS2002170 12230797

[13] Hagaman JT, Panos RJ, McCormack FX, Thakar CV, Wikenheiser-Brokamp KA, Shipley RT, Kinder BW. Vitamin D deficiency and reduced lung function in connective tissue-associated interstitial lung diseases. Chest. 2011; 139:353–60. 10.1378/chest.10-0968 20688927 PMC3032366

[14] Cutolo M. Vitamin D or hormone D deficiency in autoimmune rheumatic diseases, including undifferentiated connective tissue disease. Arthritis Res Ther. 2008; 10:123. 10.1186/ar2552 19090978 PMC2656237

[15] Deng M, Tang L, Huang D, Wang Z, Chen J. Vitamin D deficiency in connective tissue disease-associated interstitial lung disease. Clin Exp Rheumatol. 2018; 36:1049–55. 29846166

[16] Erten Ş, Şahin A, Altunoğlu A, Gemcioğlu E, Koca C. Comparison of plasma vitamin D levels in patients with Sjögren’s syndrome and healthy subjects. Int J Rheum Dis. 2015; 18:70–75. 10.1111/1756-185X.12298 24467766

[17] Zold E, Szodoray P, Gaal J, Kappelmayer J, Csathy L, Gyimesi E, Zeher M, Szegedi G, Bodolay E. Vitamin D deficiency in undifferentiated connective tissue disease. Arthritis Res Ther. 2008; 10:R123. 10.1186/ar2533 18928561 PMC2592813

[18] Toloza SM, Cole DE, Gladman DD, Ibañez D, Urowitz MB. Vitamin D insufficiency in a large female SLE cohort. Lupus. 2010; 19:13–19. 10.1177/0961203309345775 19897520

[19] Satoh M, Tanaka S, Ceribelli A, Calise SJ, Chan EK. A comprehensive overview on myositis-specific antibodies: new and old biomarkers in idiopathic inflammatory myopathy. Clin Rev Allergy Immunol. 2017; 52:1–19. 10.1007/s12016-015-8510-y 26424665 PMC5828023

[20] Ranganathan P. Genetics of bone loss in rheumatoid arthritis—role of vitamin D receptor polymorphisms. Rheumatology (Oxford). 2009; 48:342–46. 10.1093/rheumatology/ken473 19151030 PMC2722799

[21] Zhao M, Duan XH, Wu ZZ, Gao CC, Wang N, Zheng ZH. Severe vitamin D deficiency affects the expression of autophagy related genes in PBMCs and T-cell subsets in active systemic lupus erythematosus. Am J Clin Exp Immunol. 2017; 6:43–51. 28695056 PMC5498850

[22] Heulens N, Korf H, Janssens W. Innate immune modulation in chronic obstructive pulmonary disease: moving closer toward vitamin D therapy. J Pharmacol Exp Ther. 2015; 353:360–68. 10.1124/jpet.115.223032 25755208

[23] Sundar IK, Hwang JW, Wu S, Sun J, Rahman I. Deletion of vitamin D receptor leads to premature emphysema/ COPD by increased matrix metalloproteinases and lymphoid aggregates formation. Biochem Biophys Res Commun. 2011; 406:127–33. 10.1016/j.bbrc.2011.02.011 21300024 PMC3049841

[24] Ramirez AM, Wongtrakool C, Welch T, Steinmeyer A, Zügel U, Roman J. Vitamin D inhibition of pro-fibrotic effects of transforming growth factor β1 in lung fibroblasts and epithelial cells. J Steroid Biochem Mol Biol. 2010; 118:142–50. 10.1016/j.jsbmb.2009.11.004 19931390 PMC2821704

[25] Aletaha D, Neogi T, Silman AJ, Funovits J, Felson DT, Bingham CO 3rd, Birnbaum NS, Burmester GR, Bykerk VP, Cohen MD, Combe B, Costenbader KH, Dougados M, et al. 2010 Rheumatoid arthritis classification criteria: an American College of Rheumatology/European League Against Rheumatism collaborative initiative. Arthritis Rheum. 2010; 62:2569–81. 10.1002/art.27584 20872595

[26] Raghu G, Remy-Jardin M, Myers JL, Richeldi L, Ryerson CJ, Lederer DJ, Behr J, Cottin V, Danoff SK, Morell F, Flaherty KR, Wells A, Martinez FJ, et al, and American Thoracic Society, European Respiratory Society, Japanese Respiratory Society, and Latin American Thoracic Society. Diagnosis of idiopathic pulmonary fibrosis. An official ats/ers/jrs/alat clinical practice guideline. Am J Respir Crit Care Med. 2018; 198:e44–68. 10.1164/rccm.201807-1255ST 30168753

